# Soziale Ungleichheit in der regionalen Ausbreitung von SARS-CoV-2

**DOI:** 10.1007/s00103-021-03387-w

**Published:** 2021-07-23

**Authors:** Nico Dragano, Jens Hoebel, Benjamin Wachtler, Michaela Diercke, Thorsten Lunau, Morten Wahrendorf

**Affiliations:** 1 Institut für Medizinische Soziologie, Centre for Health and Society, Medizinische Fakultät, Heinrich-Heine-Universität Düsseldorf, Moorenstr. 5, 40225 Düsseldorf, Deutschland; 2 Abteilung für Epidemiologie und Gesundheitsmonitoring, Robert Koch-Institut, Berlin, Deutschland; 3 Abteilung für Infektionsepidemiologie, Robert Koch-Institut, Berlin, Deutschland

**Keywords:** Soziale Ungleichheit, COVID-19, Zeitliche Trends, Sozialepidemiologie, Deutschland, Social inequalities, COVID-19, Temporal trends, Social epidemiology, Germany

## Abstract

**Hintergrund und Ziel:**

Ob sozioökonomische Faktoren die Ausbreitung von SARS-CoV‑2 beeinflussen, ist nicht ausreichend beantwortet, da frühere Studien in der Regel kumulative Inzidenzen betrachtet und die zeitliche Entwicklung der Ausbreitung außer Acht gelassen haben. Dieser Beitrag konzentriert sich daher auf die Entwicklung von regionalen Neuinfektionen in Zusammenhang mit sozioökonomischen Faktoren. Ausgehend vom internationalen Forschungsstand präsentieren wir eigene Analysen von Meldedaten aus Deutschland.

**Methoden:**

Diese Studie untersucht regionale Daten gemeldeter COVID-19-Fälle für die 401 Landkreise und kreisfreien Städte (Kreisebene) in Deutschland und vergleicht den zeitlichen Verlauf entlang sozioökonomischer Merkmale der Kreise. Betrachtet werden altersstandardisierte wöchentliche Inzidenzen für den Zeitraum 03.02.2020–28.03.2021. Sozial- und Wirtschaftsindikatoren auf Kreisebene stammen aus der INKAR(Indikatoren und Karten zur Raum- und Stadtentwicklung)-Datenbank (z. B. Einkommen, Beschäftigtenquote, Wohnfläche).

**Ergebnisse:**

Während in der ersten und zu Beginn der zweiten Welle der Pandemie Kreise mit höherem mittleren Haushaltseinkommen höhere Inzidenzen hatten, stiegen sie in Kreisen mit niedrigem Einkommen ab Dezember 2020 deutlich an. Kreise mit einem hohen Anteil an Beschäftigten allgemein und speziell solchen im Produktionssektor hatten gerade in der zweiten und dritten Welle hohe Inzidenzen. Kreise mit einer geringen Wohnfläche je Einwohner hatten ab November 2020 ausgeprägt höhere Inzidenzen.

**Schlussfolgerung:**

Der regionale Verlauf der Pandemie unterscheidet sich nach Sozial- und Wirtschaftsindikatoren. Eine differenzierte Betrachtung dieser Unterschiede könnte Hinweise auf zielgruppenspezifische Schutz- und Teststrategien geben und helfen, soziale Faktoren zu identifizieren, die Infektionen begünstigen.

**Zusatzmaterial online:**

Zusätzliche Informationen sind in der Online-Version dieses Artikels (10.1007/s00103-021-03387-w) enthalten.

## Abstract (en)

**Background and objective:**

It has not been adequately answered whether the spread of SARS-CoV‑2 is influenced by social and economic factors. Earlier studies generally looked at cumulative incidences up to the analysis date and did not take into account the development of the spread over time. This study therefore focuses on the regional dynamic of new infections and their relationship to socioeconomic factors. Based on the literature we describe the state of knowledge and present our own analyses of administrative data from Germany.

**Methods:**

For this study, we examined regional progress data of reported COVID-19 cases for 401 cities and counties in Germany and associated them with socioeconomic characteristics of the areas. Age-standardized weekly incidence rates were calculated for the period from 3 February 2020 to 28 March 2021. Macroindicators were added from the INKAR database (e.g., income, employment rate, and crowding).

**Results:**

While areas with higher incomes and lower poverty had higher incidences in the first and at the beginning of the second wave of the pandemic, they increased significantly in low-income regions from December 2020 on. Regions with a high proportion of gainfully employed people in general and especially those in the manufacturing sector had high incidences, especially in the second wave and at the beginning of the third wave. A low mean living space per inhabitant was related to higher incidence rates since November 2020.

**Conclusion:**

The regional temporal course of the pandemic correlates with social and economic indicators. A differentiated consideration of these differences could provide information on target group-specific protection and test strategies and help to identify social factors that generally favor infections. An English full-text version of this article is available at SpringerLink as Supplementary Information.

## Einleitung

Die Dynamik der Ausbreitung von SARS-CoV‑2 („severe acute respiratory syndrome coronavirus type 2“) wird durch viele Faktoren beeinflusst. Neben den biologischen Charakteristika des Virus bestimmen beispielsweise klimatische Bedingungen, die Bevölkerungsdichte und das Alter der Bevölkerung in einem Gebiet, ihr Verhalten hinsichtlich sozialer Kontakte und Infektionsschutzmaßnahmen oder lokale Test- und Quarantänestrategien die Geschwindigkeit sowie das Ausmaß der Infektionen [1–6]. Unterscheiden sich diese Faktoren zwischen Regionen, könnten sich demnach auch die Inzidenzen unterschiedlich entwickeln. Ein systematisches Muster, das in diesem Zusammenhang von Interesse ist, ist die Schwankung der SARS-CoV-2-Infektionszahlen in Abhängigkeit von der sozioökonomischen Situation in einem Gebiet. Sozioökonomische Faktoren sind mit einer Reihe der genannten Einflussfaktoren auf das Infektionsgeschehen assoziiert (z. B. Wohnbedingungen, Bevölkerungsdichte) und könnten somit ein vorstrukturierendes Element des lokalen Infektionsgeschehens sein [7, 8].

Dass sich Infektionskrankheiten in benachteiligten Gebieten schneller verbreiten, ist bereits aus früheren Epidemien bekannt. Studien, z. B. zur saisonalen Grippe oder zum SARS-Ausbruch von 2003, berichteten in der Vergangenheit von höheren Infektionsraten in Bevölkerungen mit einer benachteiligten sozioökonomischen Position (SEP; beispielsweise gemessen durch geringe Einkommen oder Armutsquoten [8–11]). Daher wurde schon früh in der COVID-19-Pandemie vermutet, dass eine solche Ungleichheit auch in diesem Fall auftreten könnte [12, 13].

Mittlerweile wurde dieser Vermutung in verschiedenen epidemiologischen Untersuchungen nachgegangen. Die wesentliche Quelle für Daten zum Ausmaß regionaler sozioökonomischer Ungleichheiten in der COVID-19-Pandemie sind ökologische Studien, in denen Maßzahlen der Erkrankungshäufigkeit für bestimmte Gebietseinheiten (z. B. Gemeinden, Stadtteile) mit sozioökonomischen Merkmalen dieser Gebiete korreliert werden. Solche Auswertungen liegen mittlerweile aus vielen Ländern einschließlich Deutschland vor [14]. Die Befunde der vorliegenden Studien deuten in der Mehrheit an, dass benachteiligte Regionen, d. h. Regionen mit geringem Durchschnittseinkommen, hoher Einkommensungleichheit, hoher Arbeitslosigkeit oder multipler Benachteiligung (Deprivationsindizes), höhere Inzidenzen aufweisen [12, 13, 15–26]. Dieses Muster wurde sowohl für kleinräumige Vergleiche etwa von Stadtteilen [18, 22, 27] als auch für Vergleiche von größeren Gebietseinheiten etwa von Gemeinden gefunden [28, 29].

Jedoch gibt es auch gegenläufige Ergebnisse sowie Hinweise auf eine Veränderung von Zusammenhängen über die Zeit. Gerade Studien aus der frühen Phase der Pandemie im Frühjahr 2020 fanden zum Teil höhere Infektionsraten in Gebieten mit höherem Durchschnittseinkommen, höherer Bildung der Bevölkerung oder niedrigen Arbeitslosenquoten [12, 30–33]. In der Tendenz ist erkennbar, dass Untersuchungen, die später im Verlauf der Pandemie durchgeführt wurden, höhere Infektionsraten in sozioökonomisch benachteiligten Gebieten finden [27–29, 34–37]. Eine zeitliche Veränderung von höheren Fallzahlen in wohlhabenderen Gebieten hin zu höheren Fallzahlen in benachteiligten Gebieten wurde auch in Deutschland bei der Analyse von kumulativen Inzidenzen aus verschiedenen Zeiträumen im Frühjahr 2020 gefunden, was vermutlich mit anfänglichen Viruseintragungen durch sozial bessergestellte Gruppen im frühen Infektionsgeschehen in Zusammenhang steht (z. B. Skiurlauber oder Geschäftsreisende; [30, 31]).

Eine präzise Einschätzung der zeitlichen Dynamik von soziodemografischen Ungleichheiten im regionalen SARS-CoV-2-Infektionsgeschehen ist zurzeit nur schwer möglich, da fast alle durchgeführten Studien die kumulative Inzidenz von Beginn der Pandemie bis zum Zeitpunkt der Datenanalyse bzw. Periodenprävalenzen für einzelne Zeiträume als Outcome-Messung verwenden. Es gibt nur wenige Studien, die die zeitliche Dynamik der Infektionsausbreitung direkt untersuchen konnten. Dazu zählen die beiden bereits angesprochenen Studien aus Deutschland, welche Assoziationen von kumulativen Inzidenzen mit SEP-Indikatoren für verschiedene Zeiträume in der Frühphase der Pandemie verglichen. Eine weitere Analyse von Liu et al. [21] mit Daten aus dem Vereinigten Königreich hat die zeitliche Entwicklung täglicher Infektionszahlen über einen Zeitraum von 3 Monaten untersucht und ermittelt, dass ein hoher Anstieg der täglichen Fallzahlen vor allem in Regionen mit hoher sozioökonomischer Benachteiligung zu verzeichnen war. Einen ähnlichen Ansatz haben Mourad et al. [35] verfolgt und den regionalen Anstieg der Infektionszahlen im Juni 2020 in den USA als Zielgröße verwendet – mit einem ähnlichen Resultat wie die vorgenannte Studie.

Neben der Dimension der Zeit könnten auch die verwendeten Indikatoren zur Messung der sozioökonomischen Situation eine Rolle spielen. Die erwähnten umgekehrten Zusammenhänge (höhere Infektionszahlen in weniger benachteiligten Gebieten) in der Frühphase der Pandemie zeigten sich v. a. in Studien, die das Durchschnittseinkommen, den Bildungsstand oder die Arbeitslosenquote verwendeten. Bei Verwendung anderer Indikatoren wie der Armutsquote oder komplexen Deprivationsindizes, die eine Vielzahl von SEP-Indikatoren kombinieren, wurden hingegen überwiegend höhere Infektionsraten in benachteiligten Gegenden gefunden [14].

Zusammenfassend kann festgehalten werden, dass regionale Unterschiede im Infektionsgeschehen mit sozioökonomischen Merkmalen dieser Regionen in Zusammenhang stehen könnten. Bislang fehlen aber ökologische Studien, die insbesondere die zeitliche Dynamik des Infektionsgeschehens für verschiedene regionale Indikatoren der sozioökonomischen Situation betrachten. Dabei könnten solche Analysen Aufschluss darüber geben, in welchen Regionen mit kritischen Entwicklungen zu rechnen ist, wo gegebenenfalls frühzeitig zielgruppenspezifische Gegenmaßnahmen ergriffen werden sollten und ob es zeitliche Veränderungen infolge von Infektionsschutzmaßnahmen gegeben haben könnte. In diesem Beitrag stellen wir eine solche Studie mit amtlichen Meldedaten aus Deutschland vor, in der die Entwicklung der regionalen Fallzahlen in Deutschland für den Zeitraum von der Kalenderwoche 6/2020 bis zur Kalenderwoche 12/2021 (03.02.2020–28.03.2021) in Abhängigkeit von Indikatoren der sozioökonomischen Situation einer Region betrachtet wird.

## Methoden

### Datenbasis

Die hier gezeigte Auswertung ist eine ökologische Studie, bei der die Untersuchungseinheiten die 401 Landkreise und kreisfreien Städte in Deutschland sind. Die Daten zum Infektionsgeschehen stammen aus der SurvStat@RKI‑2.0‑Datenbank des Robert Koch-Instituts (RKI; Abfrage vom 01.04.2021), in der Informationen zu den durch die Gesundheitsbehörden gemäß Infektionsschutzgesetz gemeldeten laborbestätigten COVID-19-Fällen gebündelt werden. Beginnend mit Kalenderwoche 6 des Jahres 2020 (Start 03.02.2020) wurden für jede Gebietseinheit wöchentliche Inzidenzen bis zur Meldewoche 12/2021 (Ende 28.03.2021) berechnet (Fälle/Einwohnerzahl). Zur Vermeidung einer Verzerrung durch die unterschiedliche Altersstruktur der Bevölkerung in den Kreisen wurden die Inzidenzen direkt altersstandardisiert. Die überarbeitete Europastandardbevölkerung 2013 diente dabei als Referenzbevölkerung (Eurostat). Die Standardisierung erfolgte auf Basis der altersspezifischen Inzidenzen auf Kreisebene (Altersgruppierung in 5‑Jahres-Intervallen).

Die Indikatoren für die sozioökonomische Situation in den 401 Landkreisen und kreisfreien Städten stammen aus der INKAR(Indikatoren und Karten zur Raum- und Stadtentwicklung)-Datenbank des Bundesinstituts für Bau‑, Stadt- und Raumforschung (BSBR) 2021. Ausgewählt wurde zunächst das regionale Durchschnittseinkommen, das in der bisherigen Forschung bereits häufig als Indikator verwendet wurde. Es wurden 3 alternative Indikatoren verwendet: das durchschnittliche verfügbare Haushaltseinkommen aller privaten Haushalte in Euro, das Medianeinkommen pro sozialversicherungspflichtig Beschäftigten in Euro und, als Marker für den Wohlstand der Gesamtregion, das Bruttoinlandsprodukt je Einwohner (in 1000 €). Darüber hinaus wurden weitere Indikatoren verwendet. Sie wurden zum Teil noch nicht in Zusammenhang mit COVID-19 untersucht und erlauben ggf. Rückschlüsse auf weitere Determinanten von ungleichen Verteilungsmustern im Infektionsgeschehen. Untersucht wurden die mittlere Wohnfläche (in m^2^ je Einwohner), die Arbeitslosenquote (Anteil Arbeitslose an den zivilen Erwerbspersonen in %), die SGB-II-/SGB-XII-Quote (Anteil Leistungsberechtigte nach SGB II und SGB XII an allen Einwohnern in %), die Beschäftigtenquote (sozialversicherungspflichtig Beschäftigte je 100 Einwohner im Alter von 15–64) und der Anteil Erwerbstätiger an allen Erwerbstätigen im a) primären, b) sekundären und c) tertiären Sektor. Für die Analyse wurden die jeweils aktuellsten verfügbaren Daten verwendet. Diese stammen aus dem Jahr 2017, mit Ausnahme der Variablen zu den Wirtschaftssektoren (2016).

Da es sich bei den Daten um anonymisierte und räumlich aggregierte Daten handelt, war eine Vorlage bei einer Ethikkommission nicht notwendig.

### Statistische Methoden

Um den Pandemieverlauf zu untersuchen, wurden altersstandardisierte Inzidenzen je 100.000 Einwohner für jede Meldewoche im Untersuchungszeitraum betrachtet. Um mögliche Unterschiede nach SEP-Indikatoren sichtbar zu machen, sind alle Indikatoren in 3 Gruppen (niedrige, mittlere, hohe Werte) unterteilt und Inzidenzen für diese Gruppen berechnet worden. Die Unterteilung erfolgte verteilungsbasiert anhand von Terzilen. Zur Orientierung wurden in die Abbildungen zusätzlich die Daten der zentralen Interventionsmaßnahmen auf Bundesebene eingefügt (Lockdown 1 ab 22.03.2020 (LD 1)), Lockdown „light“ ab 02.11.2020 (LD light) und Lockdown 2 ab 16.12.2020 (LD 2). Neben der grafischen Darstellung wurde eine einfache Korrelationsmatrix aller verwendeten Indikatoren und der kumulierten Inzidenzen der Regionen im gesamten Beobachtungszeitraum berechnet (Pearson-Korrelationskoeffizient). Alle Berechnungen und Abbildungen erfolgten mit Stata 16.

## Ergebnisse

**Figure 1:**
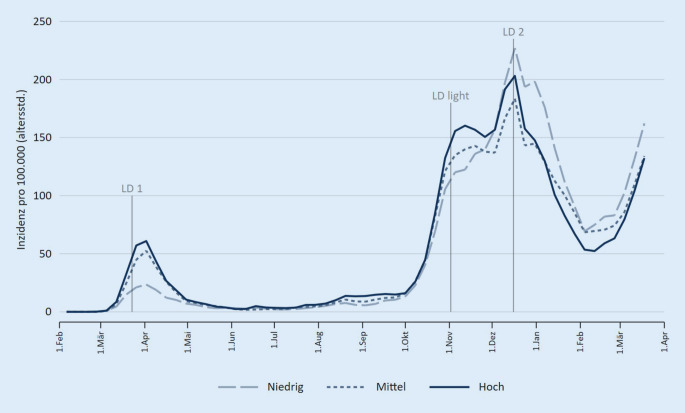

**Figure 2:**
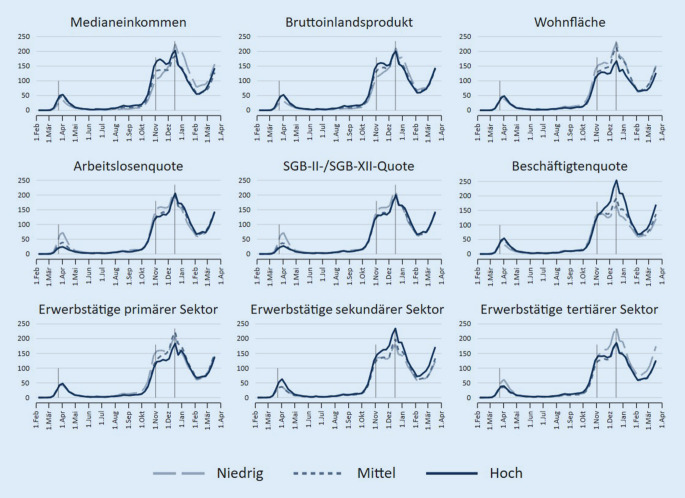

Der Verlauf der altersstandardisierten Inzidenzen in Abhängigkeit vom regionalen Haushaltseinkommen zeigt, dass in der ersten Welle der Pandemie in Deutschland im Frühjahr 2020 Kreise mit mittlerem und höherem mittleren Haushaltseinkommen stärker betroffen waren (Abb. 1). Dieses Bild zeigte sich wieder in der ersten Phase der zweiten Infektionswelle im Oktober/November 2020, kehrte sich dann aber nach der bundesweiten Verschärfung der Infektionsschutzmaßnahmen (Lockdown „light“) um. In der Folge kam es zu einem ausgeprägten Anstieg der Inzidenzen in Gebieten mit niedrigen Einkommenswerten. Die Infektionszahlen blieben dann bis zum Ende der Beobachtungszeit in Welle 3 in den Regionen mit niedrigem Einkommen gegenüber einkommensstärkeren Gebieten erhöht. Diese Muster sind auch für das Medianeinkommen der sozialversicherungspflichtig Beschäftigten erkennbar, das als alternativer Einkommensindikator in die Analysen aufgenommen wurde (Abb. 2). Für das Bruttoinlandsprodukt als Indikator für die Gesamtwirtschaftsleistung einer Region ist der Inzidenzverlauf ähnlich, die Unterschiede sind aber weniger ausgeprägt. Weiterhin sind erhöhte Inzidenzen in der zweiten und dritten Welle in Regionen mit einer niedrigen oder einer mittleren Wohnfläche je Einwohner erkennbar.

Betrachtet man mit der Arbeitslosenquote und der SGB-II-/SGB-XII-Quote jene Faktoren, die als Indikatoren für Armut in einer Region dienen können, so sind in der ersten und zu Beginn der zweiten Welle Kreise mit niedrigerer Arbeitslosigkeit und niedrigerem Umfang an Sozialleistungsbezug stärker betroffen. Dieser Trend schwächt sich im Verlauf der zweiten Welle ab und von Dezember 2020 an gibt es kaum noch Unterschiede zwischen Regionen mit hohen, mittleren oder niedrigen Arbeitslosen- bzw. SGB-II-Quoten. Diese Indikatoren hängen jedoch nicht nur mit Armut, sondern auch mit dem regionalen Arbeitsmarkt bzw. der Erwerbstätigkeit zusammen. Abb. 2 zeigt die Ausbreitung der Infektionen dann auch für die Beschäftigtenquote: Regionen, in denen diese hoch war, hatten insbesondere in der zweiten Welle höhere Inzidenzwerte. Zu allen Zeitpunkten sind hohe Inzidenzen in Kreisen mit vielen Beschäftigten im produzierenden Sektor zu verzeichnen, während ein hoher Anteil Beschäftigter im Dienstleistungssektor mit niedrigen Inzidenzen einherging.

Inwiefern die einzelnen regionalen Sozialindikatoren in Bezug auf die Entwicklung der Inzidenzen interagieren (z. B. Verzerrung der Beziehung eines Indikators mit der Infektionsentwicklung durch einen anderen Indikator (Confounding)), ist in dieser Analyse nicht zu ermitteln. Eine Annäherung erlaubt die in Tab. 1 gezeigte Korrelationsmatrix. Die Korrelationen mit der kumulativen Inzidenz im Gesamtzeitraum sind mit Vorsicht zu betrachten, weil, wie oben gezeigt wurde, zeitlich gegenläufige Zusammenhänge zu beobachten sind, die sich in der Gesamtbetrachtung aufheben. Im Wesentlichen bestätigen sich die Trends aber. Die meisten Indikatoren sind schwach bis moderat korreliert, stärkere Korrelationen bestehen zwischen den einzelnen einkommensbezogenen Faktoren und Arbeitslosigkeit/Sozialleistungen sowie zwischen den arbeitsmarktbezogenen Indikatoren.

**Table 1:** 

		1	2	3	4	5	6	7	8	9	10	11
**1**	Gesamtinzidenz	1	–	–	–	–	–	–	–	–	–	–
**2**	Haushaltseinkommen	−0,027	1	–	–	–	–	–	–	–	–	–
**3**	Medianeinkommen	−0,083	0,447	1	–	–	–	–	–	–	–	–
**4**	Bruttoinlandsprodukt	0,055	0,233	0,719	1	–	–	–	–	–	–	–
**5**	Wohnfläche	−0,298	0,196	−0,203	−0,346	1	–	–	–	–	–	–
**6**	Arbeitslosenquote	−0,054	−0,661	−0,194	−0,054	−0,391	1	–	–	–	–	–
**7**	SGB-II-/SGB-XII-Quote	−0,072	−0,617	−0,041	0,096	−0,456	0,946	1	–	–	–	–
**8**	Beschäftigtenquote	0,307	0,213	−0,248	−0,1	0,086	−0,435	−0,505	1	–	–	–
**9**	Erwerbstätige primärer Sektor	−0,126	0,048	−0,459	−0,429	0,505	−0,278	−0,399	0,292	1	–	–
**10**	Erwerbstätige sekundärer Sektor	0,271	0,18	0,028	−0,04	0,315	−0,453	−0,524	0,59	0,246	1	–
**11**	Erwerbstätige tertiärer Sektor	−0,231	−0,178	0,059	0,117	−0,389	0,476	0,565	−0,607	−0,415	−0,984	1

## Diskussion

Die hier vorgestellten Analysen zeigen, dass es Unterschiede im zeitlichen Verlauf der COVID-19-Pandemie zwischen den Kreisen in Deutschland gibt und dass diese Unterschiede mit sozioökonomischen Merkmalen dieser Kreise korrelieren. Diese explorative Studie ist weltweit eine der ersten, die über einen längeren Zeitraum die Entwicklung von regionalen Infektionszahlen in Beziehung zu sozioökonomischen Faktoren gesetzt hat. Die Ergebnisse fügen sich jedoch in die in der Einleitung zitierten bisherigen Studienergebnisse aus verschiedenen Ländern ein und bestätigen sozial differenzielle regionale Muster in der Ausbreitung von SARS-CoV‑2 einerseits und zeitliche sowie indikatorenspezifische Abweichungen andererseits.

Eine Erklärung für die gefundenen Ausbreitungsmuster ist allein auf Basis der gezeigten korrelativen Daten nicht möglich. Auch in der Literatur finden sich nur wenige direkte Untersuchungen zu möglichen Mechanismen, die zwischen regionalen sozioökonomischen Merkmalen und Infektionsrisiken vermitteln. Wahrscheinlich ist, dass die gefundenen Zusammenhänge sowohl durch individuelle Risiken der Bewohnerinnen und Bewohner bestimmter Regionen (kompositionelle Effekte) als auch durch kontextuelle Eigenschaften (kontextuelle Effekte) dieser Regionen erklärt werden. Bislang gibt es nur wenige Studien mit Individualdaten, die zudem teilweise dieselben Daten aus einer Untersuchung im Vereinigten Königreich verwendet haben (UK-Biobank-Studie). Sie betrachteten individuelle soziale Unterschiede in der Inzidenz oder der Wahrscheinlichkeit eines positiven Ergebnisses eines COVID-19-Tests [3, 26, 38–41]. Alle Studien fanden höhere Inzidenzen oder Testpositivraten bei Menschen mit niedrigerem SEP, sodass kompositionelle Effekte plausibel erscheinen, sofern Menschen mit niedrigem SEP auch gehäuft in Regionen mit niedrigem SEP auf der regionalen Ebene wohnen. Auch kontextuelle Faktoren könnten eine Rolle spielen, zu nennen ist beispielsweise die Wohnqualität, die Organisation des öffentlichen Personennahverkehrs, aber auch regionale Maßnahmen des Infektionsschutzes.

Im Folgenden möchten wir kurz beschreiben, was bislang über diese Mechanismen bekannt ist. Eine strikte Trennung zwischen Kontext und Komposition bzw. regionalen und individuellen Ungleichheiten nehmen wir dabei nicht vor, sondern orientieren uns an einem der wenigen theoretischen Modelle zur Erklärung sozialer Ungleichheiten bei Infektionskrankheiten. Das Modell von Quinn und Kumar greift zwar auf Erfahrungen früherer Pandemien zurück, bietet aber eine Systematik an, die auch bei COVID-19 hilfreich ist [8]. Die Autorinnen differenzieren vor allem 2 Prozesse: sozioökonomische Unterschiede in a) der Expositionswahrscheinlichkeit und b) in der Suszeptibilität für eine Infektion.

Die **Expositionswahrscheinlichkeit** wird bei einer Erkrankung, die vor allem im Kontakt von Mensch zu Mensch übertragen wird, durch die Anzahl, Frequenz und Dichte der sozialen Kontakte, individuelle Schutzmaßnahmen (z. B. Tragen von Masken, Hygieneverhalten) sowie Eigenschaften der Orte, an denen Kontakte stattfinden (z. B. Innenräume), bestimmt. Die sozioökonomische Position hat bekanntermaßen einen Einfluss auf diese Größen [8, 42]. Quinn und Kumar führen in diesem Zusammenhang Unterschiede in der Bevölkerungsdichte, Unterschiede im Zugang zu Basishygiene (Wasser, sanitäre Anlagen) und arbeitsbezogene Kontakte als Hauptpfade an. Alle 3 Pfade könnten auch bei SARS-CoV‑2 eine Rolle spielen.

Zunächst ist bekannt, dass die *Wohnsituation* sowohl innerhalb der eigenen Wohnung als auch im näheren Umfeld um die Wohnung mit ökonomischen Ressourcen zusammenhängt und dass ärmere Menschen häufiger in beengten Wohnverhältnissen und in Gegenden mit hoher Einwohnerdichte wohnen [42]. Zugleich sind dies Faktoren, die mit dem Infektionsgeschehen assoziiert sind: Eine hohe Einwohnerdichte in Nachbarschaften und beengte Wohnverhältnisse in den Haushalten scheinen das Infektionsrisiko mit SARS-CoV‑2 signifikant zu erhöhen [2, 43, 44], so wie es auch in dieser Analyse gefunden wurde. Ebenso gibt es Hinweise darauf, dass bauliche Unterschiede zwischen ärmeren und reicheren Gegenden bestehen und dass Einrichtungen des täglichen Lebens (z. B. Supermärkte, Restaurants) in ärmeren Stadtvierteln weniger Fläche pro Besucher haben, was das Abstandhalten („physical distancing“) erschwert [27].

Der zweite Erklärungspfad dürfte in der gegenwärtigen Situation in Deutschland einen geringeren Einfluss haben, da der *Zugang zu sauberem Wasser und sanitären Anlagen* weitgehend gegeben sein dürfte. Ausnahme ist die Situation wohnungsloser Menschen und möglicherweise auch derjenigen, die in Erstaufnahmeeinrichtungen und Gemeinschaftsunterkünften leben. Legt man diesen Aspekt jedoch weiter aus als im ursprünglichen Modell und zählt hierzu auch die Verfügbarkeit von Material zum persönlichen Infektionsschutz, wie z. B. Masken mit hoher Schutzwirkung oder Desinfektionsmittel, so könnte ein geringeres Einkommen diese Verfügbarkeit durchaus einschränken, was wiederum höhere Expositionsrisiken bedeuten könnte.

Der *Beruf* ist ein Lebensbereich, in dem eine Vielzahl sozialer Kontakte stattfindet. Naturgemäß sind in einer Pandemie Berufsgruppen mit direktem Kontakt zu Patienten:innen, Kunden:innen, Klienten:innen oder Kollegen:innen gefährdeter als Berufsgruppen, die physische Distanz halten können [45]. Allerdings ist der Zusammenhang mit der sozioökonomischen Position komplex und nicht immer eindeutig gerichtet. Zwar ist bekannt, dass Berufe mit einer hohen sozialen Kontaktrate oft auch schlechter entlohnt werden (z. B. Altenpflege, Gastronomie, einfache Produktionsberufe; [7]), allerdings ist dies nicht durchgängig so (z. B. Ärzt:innen, Lehrpersonal). Zudem waren Teile des Dienstleistungssektors während der Pandemie besonders von Betriebsschließungen betroffen, was berufliche Expositionen insgesamt reduziert haben dürfte.

Ein weiterer Aspekt ist die Möglichkeit, während einer Infektionswelle flexibel und von zu Hause aus arbeiten zu können und so Empfehlungen zur physischen Distanzierung durch die Reduzierung der Mobilität und beruflicher Kontakte umsetzen zu können. Möglichkeiten zum Homeoffice haben vor allem sozial bessergestellte Berufsgruppen mit höheren Bildungsabschlüssen und höherem Einkommen [46]. In einer statistischen Modellierung auf Basis von Daten aus großstädtischen Regionen in den USA ließen sich höhere Infektionsraten in sozial benachteiligten Gruppen darauf zurückführen, dass Personen dieser Gruppen ihre Mobilität – wahrscheinlich berufsbedingt – weniger stark einschränken konnten [27]. Relevant ist auch, mit welchem Transportmittel der Weg zur Arbeitsstätte (bzw. zur Schule oder zur Ausbildungsstätte) zurückgelegt wird. Erfolgt dies mit dem öffentlichen Nahverkehr, könnte die Wahrscheinlichkeit einer Exposition ebenfalls steigen. Hier gibt es erste Hinweise darauf, dass Menschen in ärmeren Regionen auch während der Pandemie häufiger den Personennahverkehr nutzten [16].

Im Modell von Quinn und Kumar nicht direkt angesprochen ist *das individuelle Hygiene- und Schutzverhalten*, das aber ebenfalls einen erheblichen Einfluss auf Expositionsrisiken hat. Eigenschutz setzt jedoch angemessenen Zugang zu Informationen sowie individuelle Kompetenz im Umgang mit diesen Informationen voraus – genauso wie die Bereitschaft, die Handlungsweisen dann auch auszuführen. Eine hohe Gesundheitskompetenz und Adhärenz sind wiederum mit Merkmalen wie Bildung oder Sprachkenntnissen assoziiert, sodass auch über diesen Weg ungleiche Expositionsrisiken entstehen könnten [47].

Kommt es zu einer Exposition, d. h. zu einem Erregerkontakt, wird die Frage der **Suszeptibilität**, also der Empfänglichkeit für eine Infektion, entscheidend. Auch hier machen Quinn und Kumar mögliche Unterschiede aus, die insbesondere aus der sozial ungleichen Verteilung von Risikofaktoren und Vorerkrankungen resultieren. Die sozioökonomische Ungleichheit von Krankheitsrisiken ist ein seit Langem bekanntes Phänomen der Bevölkerungsgesundheit und viele Risikofaktoren und manifeste Krankheiten treten demnach häufiger bei Menschen auf, die über weniger soziale und ökonomische Ressourcen verfügen als der Durchschnitt der Bevölkerung [7]. Das betrifft auch Risiken oder Erkrankungen, von denen angenommen wird, dass sie mit COVID-19 assoziiert sind, wie beispielsweise Rauchen, Bewegungsmangel, Übergewicht, chronischer Stress, verminderte Immunfunktionen, Herz-Kreislauf-Krankheiten oder chronische Lungenerkrankungen [7]. Direkte Untersuchungen im Kontext von SARS-CoV-2-Neuinfektionen hierzu liegen unseres Wissens jedoch nicht vor. Einzelne Untersuchungen gibt es lediglich zu schwereren Verläufen der Erkrankung, bei denen Risikofaktoren und Vorerkrankungen einen Teil des sozialen Gradienten bei schweren Verläufen erklärten [48].

In welchem Ausmaß die einzelnen Prozesse die hier gefundenen zeitlichen Muster tatsächlich beeinflusst haben, ist zum jetzigen Zeitpunkt nicht eindeutig zu beantworten. Insbesondere der Befund der Umkehr des sozialen Gradienten im Verlauf der Pandemie muss weiter untersucht werden. Jedoch scheint ein solcher Effekt nicht untypisch zu sein, wie Studien zu anderen Infektionskrankheiten nahelegen. Manabe et al. [49] untersuchten beispielsweise Patienten, die sich in der H1N1-Pandemie 2009/2010 mit der Influenza A angesteckt hatten, und verglichen solche, die in der frühen Phase der Pandemie in Mexiko infiziert wurden, mit solchen, die in späteren Phasen erkrankten. Diejenigen, die in späteren Phasen infiziert wurden, waren signifikant häufiger arm und berichteten in Interviews, dass sie kaum Zugang zu Informationen über die Erkrankung und Schutzmaßnahmen hätten. Diese Studie deutet darauf hin, dass bestimmte Infektionsschutzmaßnahmen sozial differenziell wirken. So wäre es möglich, dass in Regionen mit hoher sozioökonomischer Position Infektionsschutzmaßnahmen mit der Zeit stärkere Effekte hatten bzw. in diesen Regionen bessere Bedingungen für die Umsetzung der Maßnahmen herrschten (z. B. mehr Testzentren, bessere Aufklärung, mehr Möglichkeiten der Arbeit im Homeoffice).

In Deutschland könnte das stärkere Infektionsgeschehen in sozial bessergestellten Regionen zudem während der ersten Welle und zu Beginn der zweiten Welle ferner damit zusammenhängen, dass diese Regionen wirtschaftlich aktiver und über Wirtschaftsbeziehungen und Pendlerverflechtungen stärker miteinander im Austausch sind. Dies könnte gerade zu Beginn einer Infektionswelle, wenn Eindämmungsmaßnahmen wie Kontaktbeschränkungen noch nicht bzw. weniger stark in Kraft sind, zu höherer Mobilität und Virusübertragungen beitragen. Diese mit der Arbeit zusammenhängenden Punkte könnten die in dieser Auswertung gefundenen höheren Infektionsraten in Gebieten mit hoher Beschäftigtenquote bzw. die gegenläufigen Trends in Gebieten mit hoher, durch Arbeitslosigkeit getriebener Armut erklären. Dass Beschäftigung bedeutsam ist, legen auch die Auswertungen für die Beschäftigtenquoten nach Wirtschaftssektoren nahe. Da Produktionsunternehmen im Herbst/Winter 2020/2021 weitgehend in normalem Umfang an der Betriebsstätte tätig sein konnten, während im Dienstleistungssektor viele Betriebe geschlossen waren (z. B. Restaurants) oder ein hoher Anteil der Beschäftigten ins Homeoffice wechselte, erscheint es denkbar, dass soziale Kontakte im Arbeitskontext Inzidenzzahlen insgesamt beeinflusst haben.

Die Ergebnisse dieser Untersuchung müssen jedoch vorsichtig interpretiert werden. Die Stärke der Studie liegt in der Verwendung amtlicher Daten in Zeitreihen, ihre Schwäche in einigen methodischen Nachteilen. Die wichtigste Limitation ist das ökologische Design. Rückschlüsse auf individuelle Zusammenhänge konnten daher nicht gezogen werden. Ebenso war es nicht möglich relevante Drittvariablen (Moderatoren, Mediatoren, Confounder (Störgrößen)) wie das Geschlecht, den individuellen Gesundheitszustand oder konkurrierende regionale Einflüsse (z. B. lokale politische Eindämmungsmaßnahmen) zu berücksichtigen. Die einfache grafische Aufbereitung der Daten muss zudem in Zukunft durch komplexere statistische Methoden ergänzt werden. Damit könnten auffällige zeitliche Schwankungen der Inzidenzdaten präziser identifiziert und weitere verfügbare Indikatoren als mögliche Störgrößen berücksichtig werden (inkl. Inzidenz aus Nachbarregionen). Außerdem ist zu bedenken, dass die gewählten Gebietseinheiten (Kreise) vergleichsweise groß sind. Es ist wahrscheinlich, dass regionale Cluster eher einen kleinräumigen Charakter haben und z. B. einzelne Stadtviertel und nicht das gesamte Stadtgebiet betreffen. Es muss zudem berücksichtigt werden, dass der Outcome dieser Analyse laborbestätigte und an das RKI gemeldete COVID-19-Fälle waren. Es ist dabei von einer Untererfassung an Infektionen auszugehen, die möglicherweise abweichende sozioökonomische Verteilungsmuster aufweisen könnte. Ein weiteres Problem ist die fehlende Aktualität der Sozial- und Wirtschaftsdaten der INKAR-Datenbank, die größtenteils im Jahr 2017 erhoben wurden. Auch wenn diese Indikatoren zeitlich relativ stabil sind, ist nicht auszuschließen, dass es durch Änderungen bis zum Jahr 2020 zu Fehlklassifikationen von Gebieten gekommen sein könnte. Letztlich ist noch anzumerken, dass wir uns ausschließlich auf das Ausbruchsgeschehen bzw. die Inzidenz konzentriert haben, um Aussagen zur Dynamik der Infektion machen zu können. Weitere wichtige Aspekte wie der Schweregrad einer COVID-19-Erkrankung (inkl. Mortalität), die ebenfalls mit sozioökonomischer Benachteiligung assoziiert sind, waren nicht Gegenstand dieser Betrachtung.

## Fazit

Zusammenfassend zeigt diese Studie einen Zusammenhang zwischen der regionalen zeitlichen Dynamik der Ausbreitung von SARS-CoV‑2 und sozioökonomischen Faktoren. Die diesem Zusammenhang zugrunde liegenden Prozesse sind bislang nur rudimentär bekannt. Ein enges Monitoring, das sozial- und infektionsepidemiologische Parameter gemeinsam betrachtet, erscheint allerdings wichtig, um frühzeitig Auffälligkeiten zu erkennen. Höhere Infektionsrisiken in sozioökonomisch benachteiligten Regionen verlangen zudem eine intensivere Erforschung der Gründe sowie die Entwicklung lokaler Strategien, um besonders gefährdete Bevölkerungsgruppen gezielt zu schützen.

## Zusatzmaterial online

Englische Version des Artikels.

## References

[1] Li AY, Hannah TC, Durbin JR (2020). Multivariate analysis of black race and environmental temperature on COVID-19 in the US. Am J Med Sci.

[2] Tammes P (2020). Social distancing, population density, and spread of COVID-19 in England: a longitudinal study. BJGP Open.

[3] Jehi L, Ji X, Milinovich A (2020). Individualizing risk prediction for positive coronavirus disease 2019 testing: results from 11,672 patients. Chest.

[4] Brauner JM, Mindermann S, Sharma M (2020). Inferring the effectiveness of government interventions against COVID-19. Science.

[5] Giordano G, Blanchini F, Bruno R (2020). Modelling the COVID-19 epidemic and implementation of population-wide interventions in Italy. Nat Med.

[6] Maier BF, Brockmann D (2020). Effective containment explains subexponential growth in recent confirmed COVID-19 cases in China. Science.

[7] Bambra C, Riordan R, Ford J, Matthews F (2020). The COVID-19 pandemic and health inequalities. J Epidemiol Community Health.

[8] Quinn SC, Kumar S (2014). Health inequalities and infectious disease epidemics: a challenge for global health security. Biosecur Bioterror.

[9] Grantz KH, Rane MS, Salje H, Glass GE, Schachterle SE, Cummings DAT (2016). Disparities in influenza mortality and transmission related to sociodemographic factors within Chicago in the pandemic of 1918. Proc Natl Acad Sci USA.

[10] Bucchianeri GW (2010). Is SARS a poor man’s disease? Socioeconomic status and risk factors for SARS transmission. Forum Health Econ Policy.

[11] Chandrasekhar R, Sloan C, Mitchel E (2017). Social determinants of influenza hospitalization in the United States. Influenza Other Respir Viruses.

[12] Finch WH, Hernández Finch ME (2020). Poverty and Covid-19: rates of incidence and deaths in the United States during the first 10 weeks of the pandemic. Front Sociol.

[13] Chow DS, Soun JE, Glavis-Bloom J (2020). The disproportionate rise in COVID-19 cases among Hispanic/Latinx in disadvantaged communities of Orange County, California: a socioeconomic case-series.

[14] Wachtler B, Michalski N, Nowossadeck E (2020). Sozioökonomische Ungleichheit und COVID-19 – Eine Übersicht über den internationalen Forschungsstand. J Health Monit.

[15] Mollalo A, Vahedi B, Rivera KM (2020). GIS-based spatial modeling of COVID-19 incidence rate in the continental United States. Sci Total Environ.

[16] Sy KTL, Martinez ME, Rader B, White LF (2020). Socioeconomic disparities in subway use and COVID-19 outcomes in New York City. Epidemiol Rev.

[17] Takagi H, Kuno T, Yokoyama Y (2021). Meta-regression of COVID-19 prevalence/fatality on socioeconomic characteristics of data from top 50 U.S. large cities. J Med Virol.

[18] Whittle RS, Diaz-Artiles A (2020). An ecological study of socioeconomic predictors in detection of COVID-19 cases across neighborhoods in New York City. BMC Med.

[19] Chen JT, Krieger N (2020). Revealing the unequal burden of COVID-19 by income, race/ethnicity, and household crowding: US county versus zip code analyses. J Public Health Manag Pract.

[20] Maroko AR, Nash D, Pavilonis BT (2020). COVID-19 and inequity: a comparative spatial analysis of New York City and chicago hot spots. J Urban Health.

[21] Liu SH, Liu B, Li Y, Norbury A (2020). Time courses of COVID-19 infection and local variation in socioeconomic and health disparities in England.

[22] Baena-Díez JM, Barroso M, Cordeiro-Coelho SI, Díaz JL, Grau M (2020). Impact of COVID-19 outbreak by income: hitting hardest the most deprived. J Public Health (Oxf).

[23] Das A, Ghosh S, Das K, Basu T, Das M, Dutta I (2020). Modeling the effect of area deprivation on COVID-19 incidences: a study of Chennai megacity, India. Public Health.

[24] Lamb MR, Kandula S, Shaman J (2020). Differential COVID-19 case positivity in New York City neighborhoods: socioeconomic factors and mobility. Influenza Other Respir Viruses.

[25] Lieberman-Cribbin W, Tuminello S, Flores RM, Taioli E (2020). Disparities in COVID-19 testing and positivity in New York City. Am J Prev Med.

[26] Rozenfeld Y, Beam J, Maier H (2020). A model of disparities: risk factors associated with COVID-19 infection. Int J Equity Health.

[27] Chang S, Pierson E, Koh PW (2020). Mobility network models of COVID-19 explain inequities and inform reopening. Nature.

[28] Moise IK (2020). Variation in risk of COVID-19 infection and predictors of social determinants of health in Miami-Dade County, Florida. Prev Chronic Dis.

[29] Ginsburgh V, Magerman G, Natali I (2021). COVID-19 and the role of inequality in French regional departments. Eur J Health Econ.

[30] Wachtler B, Michalski N, Nowossadeck E (2020). Sozioökonomische Ungleichheit im Infektionsrisiko mit SARS-CoV-2 – Erste Ergebnisse einer Analyse der Meldedaten für Deutschland. J Health Monit.

[31] Plümper T, Neumayer E (2020). The pandemic predominantly hits poor neighbourhoods? SARS-coV-2 infections and Covid-19 fatalities in German districts. Eur J Public Health.

[32] Abedi V, Olulana O, Avula V (2020). Racial, economic, and health inequality and COVID-19 infection in the United States. J Racial Ethn Health Disparities.

[33] Mukherji N (2020). The social and economic factors underlying the incidence of COVID-19 cases and deaths in US counties.

[34] Khanijahani A (2021). Racial, ethnic, and socioeconomic disparities in confirmed COVID-19 cases and deaths in the United States: a county-level analysis as of November 2020. Ethn Health.

[35] Mourad A, Turner NA, Baker AW (2020). Social disadvantage, politics, and SARS-coV-2 trends: a county-level analysis of United States data. Clin Infect Dis.

[36] Richmond HL, Tome J, Rochani H, Fung IC-H, Shah GH, Schwind JS (2020). The use of penalized regression analysis to identify county-level demographic and socioeconomic variables predictive of increased COVID-19 cumulative case rates in the state of Georgia. Int J Environ Res Public Health.

[37] Palacio A, Tamariz L (2020). Social determinants of health mediate COVID-19 disparities in south Florida. J Gen Intern Med.

[38] Chadeau-Hyam M, Bodinier B, Elliott J (2020). Risk factors for positive and negative COVID-19 tests: a cautious and in-depth analysis of UK biobank data. Int J Epidemiol.

[39] McQueenie R, Foster HME, Jani BD (2020). Multimorbidity, polypharmacy, and COVID-19 infection within the UK biobank cohort. PLoS ONE.

[40] Ho FK, Celis-Morales CA, Gray SR (2020). Modifiable and non-modifiable risk factors for COVID-19: results from UK Biobank.

[41] Vahidy FS, Nicolas JC, Meeks JR (2020). Racial and ethnic disparities in SARS-CoV-2 pandemic: analysis of a COVID-19 observational registry for a diverse US metropolitan population. BMJ Open.

[42] Blumenshine P, Reingold A, Egerter S, Mockenhaupt R, Braveman P, Marks J (2008). Pandemic influenza planning in the United States from a health disparities perspective. Emerg Infect Dis.

[43] Millett GA, Jones AT, Benkeser D (2020). Assessing differential impacts of COVID-19 on black communities. Ann Epidemiol.

[44] Ahmad K, Erqou S, Shah N (2020). Association of poor housing conditions with COVID-19 incidence and mortality across US counties. PLoS ONE.

[45] Möhner M, Wolik A (2020). Berufs- und branchenbezogene Unterschiede im COVID-19-Risiko in Deutschland. Dtsch Arztebl Int.

[46] von Gaudecker H-M, Holler R, Janys L (2020). Labour supply in the early stages of the COVID-19 pandemic: empirical evidence on hours, home office, and expectations.

[47] McCormack LA, Squiers L, Frasier AM, Lynch M, Bann CM, MacDonald PDM (2021). Gaps in knowledge about COVID-19 among US residents early in the outbreak. Public Health Rep.

[48] Williamson EJ, Walker AJ, Bhaskaran K (2020). Factors associated with COVID-19-related death using OpenSAFELY. Nature.

[49] Manabe T, Higuera Iglesias AL, Vazquez Manriquez ME (2012). Socioeconomic factors influencing hospitalized patients with pneumonia due to influenza A(H1N1)pdm09 in Mexico. PLoS One.

