# Supplementary material for: Social inequalities in the regional spread of SARS-CoV-2 infections
Source: Bundesgesundheitsblatt Gesundheitsforschung Gesundheitsschutz. 2021 Jul 23;64(9):1116–24. [Article in German] doi: 10.1007/s00103-021-03387-w (PMC8298974; doi:10.1007/s00103-021-03387-w)
Supplement: Supplementary file 1 — Englische Version des Artikels. [file 103_2021_3387_MOESM1_ESM.pdf]

Bundesgesundheitsblatt 9/2021

*Electronic Supplementary Material: English version of the article “**Soziale Ungleichheit in der regionalen Ausbreitung von SARS-CoV-2**”*

Social inequalities in the regional spread of SARS-CoV-2

Nico Dragano[1], Jens Hoebel[2], Benjamin Wachtler[2], Michaela Diercke[3], Thorsten Lunau[1], Morten Wahrendorf[1]

[1] Institute of Medical Sociology, Centre for Health and Society, Medical Faculty and University Hospital, University of Düsseldorf, Düsseldorf, Germany

[2] Robert Koch Institute, Department of Epidemiology and Health Monitoring, Berlin, Germany

[3] Robert-Koch-Institute, Department of Infectious disease, Berlin, Germany

Corresponding Autor:

Prof. Dr. Nico Dragano

Institute of Medical Sociology

Medical Faculty

Heinrich-Heine-University Düsseldorf

Moorenstraße 5

40225 Düsseldorf, Germany

dragano@med.uni-duesseldorf.de

Abstract

Background and Objective

It is still unknown if the spread of SARS-CoV-2 is influenced by social and economic factors. Earlier studies generally looked at cumulative incidences across time without studying trajectories of incidences over time, and thus, developments of the spread. This study, therefore, focuses on the regional dynamics of new infections and their relationship to socio-economic characteristics. Based on the literature, we describe the state of knowledge and present our own analysis of administrative data from Germany.

Methods

We use notification data of COVID-19 cases for 401 cities and counties from February 3, 2020 till March 28, 2021 in Germany and associate them with socio-economic characteristics of the areas. Age-standardised weekly incidence rates were calculated and macro indicators were added from the INKAR database (e.g. income, social benefit rate, employment rate, overcrowding).

Results

While areas with higher incomes and lower poverty had higher incidences during the first and at the beginning of the second wave of the pandemic, from December 2020 onwards incidence rates were generally higher in low-income regions. Regions with high employment rates (especially if employed in the manufacturing sector) had high incidences, particularly in the second wave and at the beginning of the third wave. Regions with lower average living space also had higher incidence rates since November 2020.

Conclusion

Regional trajectories of incidences are associated with social and economic indicators. This finding could provide information on target group-specific protection and test strategies and help to identify social factors that are linked to increased infection rates.

Keywords

Health inequalities, Social Inequalities, COVID-19, Social epidemiology, Germany

Introduction

The dynamics of the spread of SARS-CoV-2 (severe acute respiratory syndrome coronavirus type 2) are influenced by many factors. In addition to the biological characteristics of the virus, other factors determine the speed and spread of infections, for example, climatic conditions, population density and age of the population in a geographic area, the behaviour with regard to social contacts and infection protection measures or local test and quarantine strategies [1–6]. If these factors differ between regions, different incidence rates may result. A systematic pattern of interest in this context is the fluctuation in the number of SARS-CoV-2 infections depending on socio-economic characteristics of a geographic area. Socio-economic factors are associated with a number of the influencing factors on infection process (e.g. living conditions, population density) and could thus be a pre-structuring element of the local infection process [7, 8].

The fact that infectious diseases spread faster in disadvantaged areas is already known from earlier epidemics. Studies on seasonal influenza or the SARS outbreak in 2003, for instance, report higher infection rates in populations with a disadvantaged socio-economic position (SEP; which is mostly measured by low income or poverty rates) [8–11]. Therefore, already early on in the COVID-19 pandemic, it was suspected that such inequality could also occur in this pandemic [12, 13].

This assumption has now been followed in various epidemiological investigations. The most essential source currently of the regional extent of socio-economic inequalities in the COVID-19 pandemic are ecological studies.Here measures of the incidence of the disease for specific territorial units (e.g. communities, districts) are correlated with socio-economic characteristics of these areas. Such evaluations are now available from many countries, including Germany [14]. Overall, the findings of the available studies indicate that disadvantaged regions, in terms of low average income, high-income inequality, high unemployment or multiple disadvantages (deprivation indices), have a higher incidence [12, 13, 15–26]. This was found for small-area comparisonssuch as city districts [18, 22, 27], and large-areas like municipalities [28, 29].

However, there are also studies with contradictory results and indications of changing relationships between factors over time. Especially studies from the early phase of the pandemic in spring 2020 found higher infection rates in areas with a higher average income, higher education of the population or lower unemployment rates [12, 30–33]. There is a noticeable tendency that studies carried out later in the course of the pandemic find higher infection rates in socio-economically disadvantaged areas [27–29, 34–37]. A temporal change from higher case numbers in more affluent areas to higher case numbers in disadvantaged areas was also found in the analysis of cumulative incidences from different periods in spring 2020 in Germany, and is

probably related to initial virus entries by socially better-off groups during the early phase of the pandemic (e.g. ski holidays or business travellers) [30, 31].

A precise assessment of the dynamics of socio-demographic inequalities in the regional SARS-CoV-2 infection process over time is currently missing because most studies used cumulative incidence from the beginning of pandemic to the point of data analysis or periodical prevalence for selected time periods outcome measurements. There are only a few studies that were able to directly examine the temporal dynamics of the spread of infection. These include the two studies from Germany already mentioned, which compared associations of cumulative incidences with SEP indicators for different time periods at the beginning of the pandemic. An analysis by Liu et al. [21] with data from England examined the temporal development of the daily number of infections over a period of three months and determined that a high increase in the daily number of cases was recorded, especially in regions with high socio-economic disadvantage. Mourad et al. [35] pursued a similar approach and used the regional increase in the number of infections in June 2020 in the USA as a target – with a similar result as the previously mentioned study.

In addition to time, the socio-economic situation could play a role. The mentioned higher infection rates in less disadvantaged areas at the beginning of the pandemic have been shown in studies using average income, level of education or unemployment rate. When using other indicators such as poverty rate or complex deprivation indices that integrate a large number of SEP indicators, higher infection rates were found in disadvantaged areas [14].

In summary, it can be stated that regional differences in the infection rate could be related to the socio-economic characteristics of these regions. So far, there is also a lack of ecological studies, which consider specifically the temporal dynamics of the infection process for various regional indicators of social-economic situation. Such analyses could provide information about regions where critical developments could be expected and group-specific interventions should be performed. In the present article, we present such a study using official notification data from Germany in which the development of the regional number of cases in Germany for the period from calendar week 6/2020 to calendar week 12/2021 (03.02.2020-28.03.2021) is considered depending on indicators of the socio-economic situation of a region.

Methods

Database

The analysis shown here is an ecological study in which the investigation units are the 401 rural districts and urban districts in Germany. The data on the incidence of infection come from the SurvStat@RKI 2.0 database of the Robert Koch Institute (data access 01. April 2021), which provides information on the laboratory-confirmed COVID-19 cases notified to the health authorities in accordance with the Infection Protection Act. Starting with calendar week 6 of 2020 (start 03.02.2020), weekly incidences up to notification week 12/2021 (end 28.03.2021) were calculated for each regional unit (cases/population). To avoid a bias due to the different age structure of the population in the districts, the incidences were directly age-standardised. The revised European standard population 2013 was used as the reference population (Eurostat). The standardisation was carried out based on the age-specific incidences at the district level (age grouping in five-year intervals).

The indicators for the socio-economic situation in the 401 rural districts and urban districts come from the INKAR database of the Federal Institute for Building, Urban and Spatial Research (BSBR 2021). First of all, regional average income was selected, which has often been used as an indicator in previous research. Three alternative indicators were used: the average disposable household income of all private households in Euro, the mean income for employees subject to social insurance contributions in Euro and, as a marker for the prosperity of the region as a whole, the gross domestic product per inhabitant (in 1,000 Euro). Other indicators were also used. Some of them have not yet been investigated together with COVID-19 and may allow additional conclusions to be drawn about determinants of unequal distribution patterns in the infection process. The average living space (in m^2 per inhabitant), the unemployment rate (proportion of the unemployed in the labour force in %), the proportion of the population receiving social benefit payments (according to the German Social Code, SGB II / XII), the employment rate (labour force per 100 inhabitants of working age, i.e. 15-64 years) and the proportion of workers of all working persons in the a) primary, b) secondary and c) tertiary sectors. The most recent data available were used for the analysis. These are from 2017, except for the variables on the economic sectors (2016).

Since the data are anonymous and spatially aggregated, a submission to an ethics committee was not necessary.

Statistical methods

In order to examine the course of the pandemic, courses for the age-standardised incidences per 100,000 inhabitants for each notification week in the course of the study were considered.

In order to show possible differences according to SEP indicators, all indicators were divided into three groups (low-medium-high values) and incidence rates were calculated for these groups. The dates of core intervention measures at the federal level in Germany have also been included in the figures (Lockdown 1 (March 22, 2020 (LD 1)), Lockdown "light" (November 2, 2020 (LD light)) and Lockdown 2 (December 16, 2020 (LD 2)). In addition to the graphical representation, a simple correlation matrix of all indicators used and the cumulative incidences of the regions in the entire observation period was calculated (Pearson correlation coefficient). All calculations and figures were produced with Stata 16.

Results

The course of the age-standardised incidences as a function of regional household income shows that in the first wave of the epidemic in Germany in spring 2020, districts with middle and higher average household income were more severely affected (Figure 1). This picture emerged again in the first phase of the second wave of infections in October/November 2020, but was then reversed after the nationwide implementation of new infection protection measures, called "Lockdown light", when there was a marked increase in incidences in low-income areas. The number of infections then remained higher in the regions with low income compared to higher-income regions until the end of the observation period. These patterns can also be seen for the median income included in the analyses as an alternative income indicator (Figure 2). The incidence curve is also similar for the gross domestic product (GDP), as an indicator of the overall economic performance of a region, but the differences are less pronounced. Higher incidences in the second and third wave can, however, be identified for regions with low or medium living space per inhabitant.

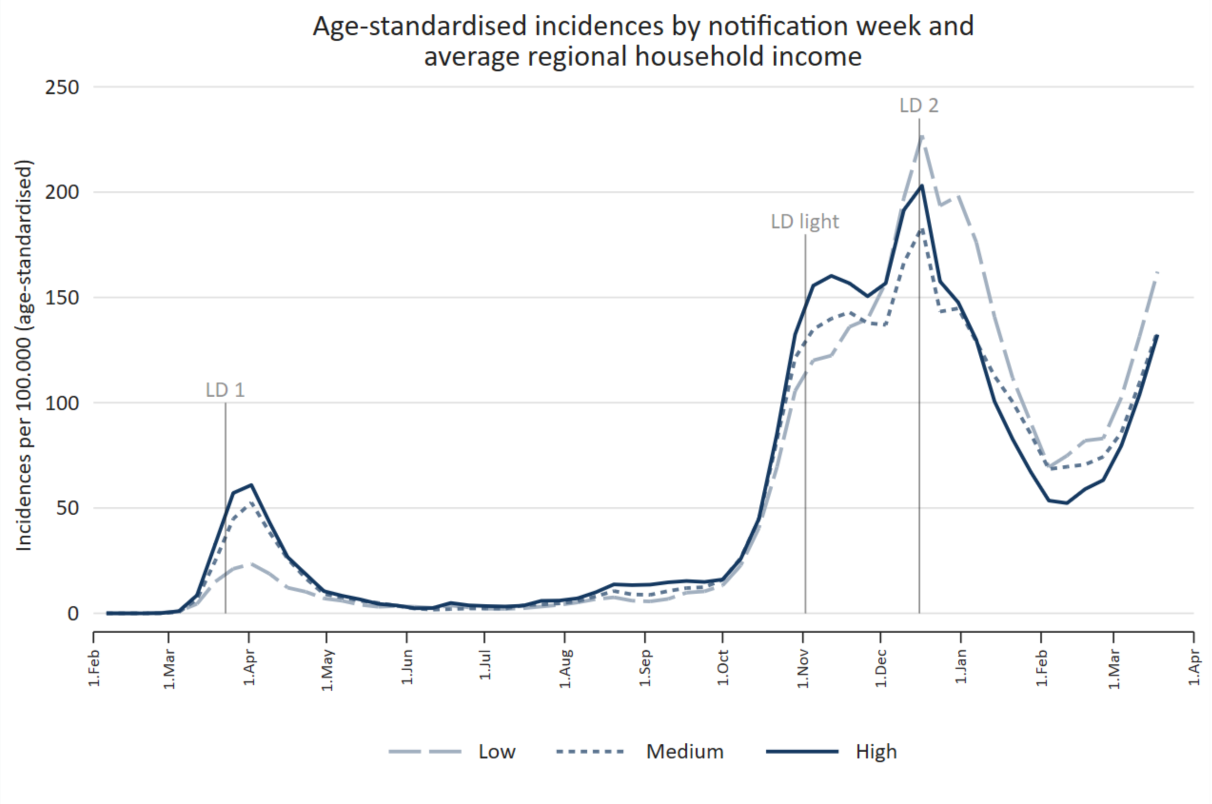

Figure 1: Development of the age-standardised regional incidences (districts, n=401) per notification week in the study period depending on the regional average household income

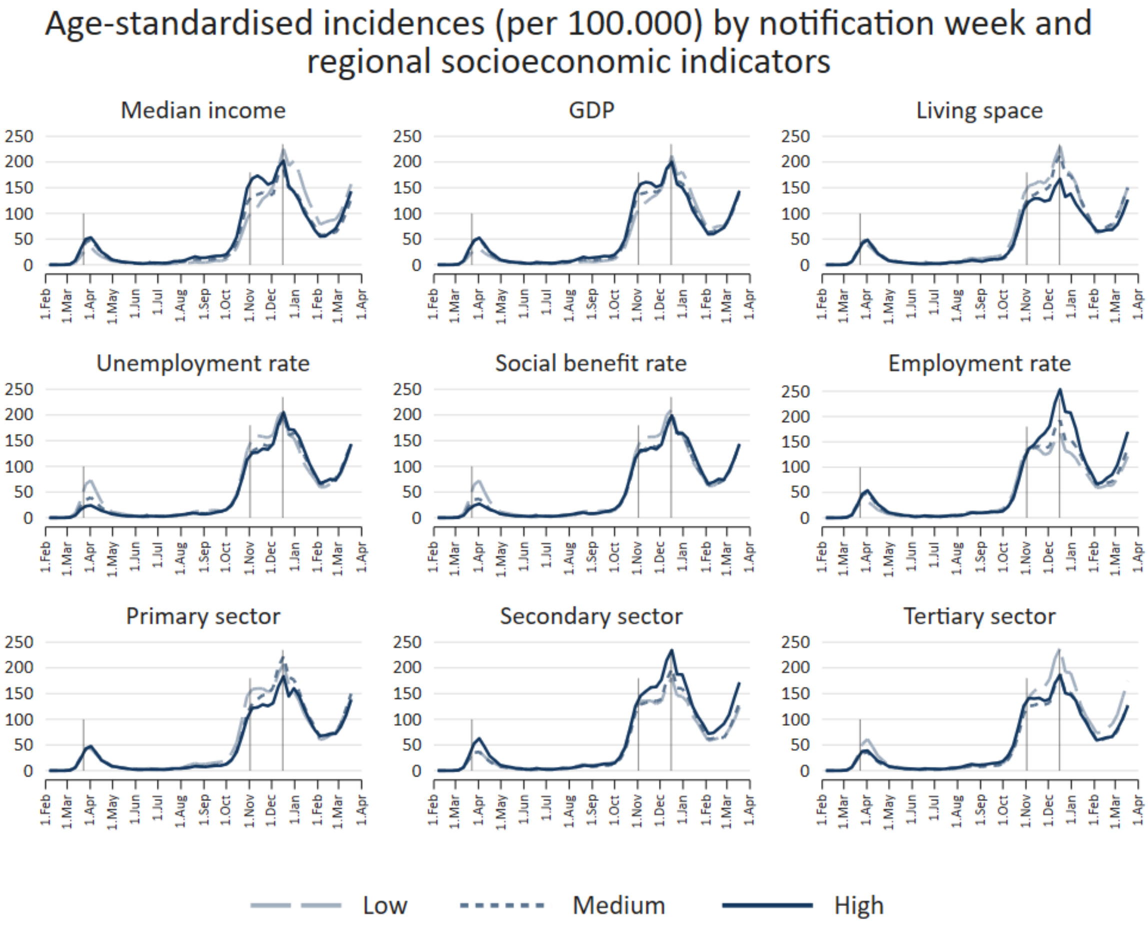

Figure 2: Development of the age-standardised regional incidences (districts, n=401) per notification week in the study period depending on the characteristics of various socio-economic indicators

If the unemployment rate and the Social Code (SGB II) rate are considered as factors that can serve as indicators of poverty in a region, in the first and at the beginning of the second wave, districts with lower unemployment and lower level of social benefit receipt were more affected. However, this trend weakened in the course of the second wave and from December 2020 onwards are hardly any differences between regions with high, medium or low unemployment or Social Code (SGB II) rates were noted. However, these indicators are not only related to poverty but also to the regional labour market and employment. Figure 2 shows the spread of infections for the employment rate: Regions, where this rate was high, had higher incidence rates, especially during the second pandemic wave. At all times, high incidences were recorded in districts with many employees in the secondary sector, while low incidences were seen in distritics with a high proportion of employees in the tertiary sector.

The extent to which the individual regional social indicators interact with regard to the development of incidences (e.g. confusion of the relationship of one indicator with the development of infection by another indicator) cannot be clearly determined in this analysis. The correlation matrix shown in Table 1 allows an approximation. The correlations with the cumulative incidence in the total period should be viewed with caution because, as shown above, temporal correlations in opposite directions can be observed, which cancel each other out in the overall consideration. Essentially, however, the trends are confirmed. Most indicators are weakly to moderately correlated, stronger correlations exist between the individual income-related factors and unemployment/social benefits, as well as between the labour market-related indicators.

Table 1: Pearson correlation coefficients for all socio-economic-indicators on district level

		-1	-2	-3	-4	-5	-6	-7	-8	-9	-10	-11
-1	Overall incidence	1										
-2	Household income	-0.027	1									
-3	Median income	-0.083	0.447	1								
-4	GDP	0.055	0.233	0.719	1							
-5	Living space	-0.298	0.196	-0.203	-0.346	1						
-6	Unemployment rate	-0.054	-0.661	-0.194	-0.054	-0.391	1					
-7	Social benefit rate	-0.072	-0.617	-0.041	0.096	-0.456	0.946	1				
-8	Employment rate	0.307	0.213	-0.248	-0.1	0.086	-0.435	-0.505	1			
-9	Primary sector	-0.126	0.048	-0.459	-0.429	0.505	-0.278	-0.399	0.292	1		
-10	Secondary sector	0.271	0.18	0.028	-0.04	0.315	-0.453	-0.524	0.59	0.246	1	
-11	Tertiary sector	-0.231	-0.178	0.059	0.117	-0.389	0.476	0.565	-0.607	-0.415	-0.984	1

Discussion

The analyses presented here show that there are differences over time in the course of the COVID-19 epidemic between the districts in Germany and that these differences correlate with socio-economic characteristics of these districts. This study, is among the first studies world-wide to relate the development of regional infection rates to socio-economic factors over a longer period of time. The results are in line with previous study results from various countries and confirm socially differential regional patterns in the spread of SARS-CoV-2, including temporal and indicator-specific deviations.

An empirical explanation for the patterns found is not possible on the basis of the correlative data shown. Also, in the literature, there are only a few direct studies of possible mechanisms that mediate between regional socio-economic characteristics and the risk of infection. It is likely that the observed correlations are explained both by individual risks of the inhabitants of certain regions (compositional effects) and by contextual characteristics (contextual effects) of these regions. To date, there are only a few studies with individual data, mostly based on UK Biobank data. These studies investigated individual social differences in the incidence or probability of a positive result of a COVID-19 test [3, 26, 38–41]. All studies found higher incidences or positive test rates among people with disadvantaged SEP, so compositional effects seem plausible if people with disadvantaged SEP live more often in regions that are socio-economically disadvantaged regions. Additionally, contextual factors such as the quality of living, the organisation of local public transport and the regional infection control measures could play a role as well.s.

In the following, we would like to describe what is known about these mechanisms. We do not make a strict separation between the mentioned contextual and compositional effects or regional and individual inequalities. Instead, we rely on one of the few theoretical models to explain social inequalities in infectious diseases. This model was developed by Quinn and Kumar and was based on experiences from previous pandemics. It offers a systematic approach that is useful also during the COVID-19 pandemic [8]. Concerning infection risks, the authors differentiate between two main processes: socio-economic differences in a) the probability of exposure and b) in the susceptibility to infection.

In the case of a disease that is mainly transmitted through human-to-human contact, the probability of exposure is determined by the number, frequency and density of social contacts, individual protective measures (e.g. wearing masks, hygienic behaviour), and the characteristics of the places where contacts take place (e.g. interiors). The socio-economic position is known to have an influence on these variables [8, 42]. In this context, Quinn and Kumar cite differences in population density, differences in access to basic hygiene (e.g. water, sanitation)

and work-related contacts as the main paths. All three paths could also play a role in SARS-CoV-2.

First of all, it is known that the **living situation** is related to economic resources both within one's own home and in the immediate vicinity of the home [42]. It is also known that poorer people are more likely to live in cramped living conditions and in areas with a high population density [42]. At the same time, these factors are associated with the infection process: A high population density in neighbourhoods and cramped living conditions in households seem to increase the risk of infection with SARS-CoV-2 significantly [2, 43, 44], as was found in this analysis. There are also indications that there are structural differences between poorer and richer areas and that facilities for everyday life (e.g. supermarkets, restaurants) in poorer districts have less space per visitor, making *physical distancing* difficult [27].

The second path of explanation is likely to have less of an impact in the current situation in Germany, since **access to clean water and sanitation** should largely be given. However, homeless people or people in initial reception facilities and collective accommodation, maybe exceptions to this. However, if this aspect is interpreted more broadly than in the original model and this also includes the availability of material for personal protection against infection, such as masks with a high protective effect or disinfectants, then a lower income could definitely limit this availability, which in turn could mean higher exposure risks.

Work is an area of life in which a large number of social contacts take place. Naturally, in a pandemic, professional groups with direct contact to patients, customers, clients or colleagues are at greater risk than professional groups that are able to maintain a physical distance [45]. However, the connection with the socio-economic position is complex and not always clearly established. It is known that essential workers with a high social contact rate are often poorly paid (e.g. care for the elderly, gastronomy, simple production jobs) [7], although this is not always the case (e.g. doctors, teaching staff). In addition, parts of the tertiary sector were particularly affected by business closures during the pandemic, which is likely to have reduced occupational exposure overall.

Another aspect is the possibility of being able to work flexibly and from home during a wave of infection and, therefore, to be able to implement recommendations on physical distancing by reducing mobility and professional contacts. Socially better-off occupational groups with higher educational qualifications and higher incomes have better opportunities to work from home [46]. In a statistical model based on data from metropolitan regions in the USA, higher infection rates in socially disadvantaged groups could be attributed to the fact that people in these groups were less able to restrict their mobility [27]. Furthermore, also the means of transport to get to work (or to school or training centre) is relevant. If this is done by local public transport,

the likelihood of exposure could also increase. There are initial indications that people living in poorer regions used local public transport more frequently, even during the pandemic [16].

Although Individual hygiene and protective behaviour are not directly addressed in the model by Quinn and Kumar, they play a significant role on exposure risks. Self-protection requires adequate access to information, as well as individual competence in handling this information – as well as the willingness to carry out the actions. A high level of health literacy and adherence is in turn associated with characteristics such as education or language skills. Thus, unequal exposure risks could arise as well [47].

If there is exposure, i.e. contact with the pathogen, the question of susceptibility (i.e. susceptibility to infection), becomes decisive. Quinn and Kumar also identify possible differences resulting from the socially unequal distribution of risk factors and previous illnesses. The socio-economic inequality of disease risks is a long-known phenomenon of the health of the population and many risk factors as well as chronic diseases are therefore more common in people, who have fewer social and economic resources than the average population [7]. This also applies to risks or diseases that are assumed to be associated with COVID-19 and severe disease courses, such as smoking, sedentary lifestyle, obesity, chronic stress, decreased immune functions, cardiovascular diseases or chronic lung diseases [7]. However, to the best of our knowledge, there are no direct investigations in the context of new SARS-CoV-2 infections. Studies on an individual level are only available on more severe courses of the disease, in which risk factors and previous illnesses explained part of the social gradient in severe disease courses [48].

The extent to which the individual processes actually influenced the temporal patterns found here cannot be clearly answered at this point. In particular, findings of the reversed social gradient in the course of the pandemic requires more research. However, this reversal does not seem to be atypical, as studies on other infectious diseases suggest. Manabe et al. [49], for example, investigatedpatients who were infected with influenza A in the 2009/2010 H1N1 pandemic and compared those who were infected in the early phase of the pandemic in Mexico with those who became ill in later phases. Those who were infected in later phases were significantly more likely to be poor and reported in interviews that they had little access to information about the disease and protective measures. This study suggests that specific measures for infection protection may affect groups differently. Possibly, socio-economically more advantaged regions are more successful in implementing control measures with time (e.g. more test centres, more opportunities to work from home).

In Germany, the higher rate of infection in socially better-off regions during the first wave and at the beginning of the second wave could be related, among other things, to the fact that these regions are more economically active and are in greater contact with one another by means of

economic relationships and commuter ties. Especially at the beginning of a wave of infections, when containment measures such as contact restrictions are not yet in place or only limited, this could contribute to higher mobility and virus transmissions. These work-related points could explain the higher infection rates found in this analysis in areas with a high employment rate or opposing trends in areas with high unemployment-driven poverty. The evaluations for the employment rates by the economic sector also suggest that employment is important. Since production companies were largely able to work at the business premises to the normal extent in autumn/winter 2020/2021, while many businesses in the tertiary sector were closed (e.g. restaurants) or a high proportion of employees switched to work from home, it seems conceivable that social contacts have influenced overall incidence figures in the work context.

However, the results of this investigation have to be interpreted with caution. The strength of the study include the use of official time-series data. Weaknesses that should be considered concern primarily methodological limitations. The most important limitation is the ecological design. Conclusions on an individual level remain not possible. Likewise, it was not possible to take into account confounders such as gender, individual health status or competing regional influences (e.g. local political containment measures). Our study must also be supplemented by more complex statistical methods in the future. It should also be noted that the selected territorial units (districts) are too large to allow a more precise localisation of affected areas. It is likely that spatial differences in disease rates tend to have a small-scale character and, for example, affect single urban districts and not the entire urban area. It must also be taken into account that the outcomes of this analysis were laboratory-confirmed COVID-19 cases reported to the RKI. It can be assumed that infections are under-recorded, which could possibly show different socio-economic distribution patterns. Another problem is that more recent social and economic data from the INKAR database would have been desirable. Even if these indicators are relatively stable over time, it cannot be ruled out that changes since 2017 could lead to misclassifications.

Finally, it should be noted that we have only focused on the incidence in order to be able to make statements about the dynamics of the infection. Other important aspects, such as the severity of a COVID-19 disease (including mortality), which are also associated with socio-economic disadvantage, were not the subject of this analysis.

In summary, this study shows a connection between the regional temporal dynamics of the spread of SARS-CoV-2 and socio-economic factors. The processes underlying this relationship are still not clear. However, close monitoring that considers social and infection epidemiological parameters together appears to be important in order to recognise abnormalities at

an early stage. Higher infection risks in socio-economically disadvantaged regions also require more intensive research into the reasons and the development of local strategies to specifically protect vulnerable population groups.

Conflict of interest

The authors declare that there are no conflicts of interest.

Note regarding funding

This work was funded by the German Research Foundation (DFG) (project numbers: HO 6565/2-1 and WA 3065/64-1). In addition, it was developed in the context of the MethodCoV method platform, which was funded by the Federal Ministry of Education and Research (BMBF) under the funding code *01KX2021* .

References

1. Li AY, Hannah TC, Durbin JR et al. (2020) Multivariate Analysis of Black Race and Environmental Temperature on COVID-19 in the US. Am J Med Sci 360:348–356. https://doi.org/10.1016/j.amjms.2020.06.015
2. Tammes P (2020) Social distancing, population density, and spread of COVID-19 in England: a longitudinal study. BJGP Open 4. https://doi.org/10.3399/bjgpopen20X101116
3. Jehi L, Ji X, Milinovich A et al. (2020) Individualizing Risk Prediction for Positive Coronavirus Disease 2019 Testing: Results From 11,672 Patients. Chest 158:1364–1375. https://doi.org/10.1016/j.chest.2020.05.580
4. Brauner JM, Mindermann S, Sharma M et al. (2020) Inferring the effectiveness of government interventions against COVID-19. Science 371:6531. https://doi.org/10.1126/science.abd9338
5. Giordano G, Blanchini F, Bruno R et al. (2020) Modelling the COVID-19 epidemic and implementation of population-wide interventions in Italy. Nat Med 26:855–860. https://doi.org/10.1038/s41591-020-0883-7
6. Maier BF, Brockmann D (2020) Effective containment explains subexponential growth in recent confirmed COVID-19 cases in China. Science 368:742–746. https://doi.org/10.1126/science.abb4557
7. Bambra C, Riordan R, Ford J, Matthews F (2020) The COVID-19 pandemic and health inequalities. J Epidemiol Community Health 74:964–968. https://doi.org/10.1136/jech-2020-214401
8. Quinn SC, Kumar S (2014) Health inequalities and infectious disease epidemics: a challenge for global health security. Biosecur Bioterror 12:263–273. https://doi.org/10.1089/bsp.2014.0032
9. Grantz KH, Rane MS, Salje H, Glass GE, Schachterle SE, Cummings DAT (2016) Disparities in influenza mortality and transmission related to sociodemographic factors within Chicago in the pandemic of 1918. Proceedings of the National Academy of Sciences of the United States of America 113:13839–13844. https://doi.org/10.1073/pnas.1612838113
10. Bucchianeri GW (2010) Is SARS a Poor Man's Disease? Socioeconomic Status and Risk Factors for SARS Transmission. Forum for Health Economics & Policy 13:115. https://doi.org/10.2202/1558-9544.1209
11. Chandrasekhar R, Sloan C, Mitchel E et al. (2017) Social determinants of influenza hospitalization in the United States. Influenza Other Respir Viruses 11:479–488. https://doi.org/10.1111/irv.12483
12. Finch WH, Hernández Finch ME (2020) Poverty and Covid-19: Rates of Incidence and Deaths in the United States During the First 10 Weeks of the Pandemic. Front. Sociol. 5. https://doi.org/10.3389/fsoc.2020.00047
13. Chow DS, Soun JE, Glavis-Bloom J et al. (2020) The disproportionate rise in COVID-19 cases among Hispanic/Latinx in disadvantaged communities of Orange County, California: A socioeconomic case-series, 129 Suppl 2
14. Wachtler B, Michalski N, Nowossadeck E et al. (2020) Sozioökonomische Ungleichheit und COVID-19 – Eine Übersicht über den internationalen Forschungsstand. Journal of Health Monitoring 5:3–18. https://doi.org/10.25646/7058
15. Mollalo A, Vahedi B, Rivera KM (2020) GIS-based spatial modeling of COVID-19 incidence rate in the continental United States. Sci Total Environ 728:138884. https://doi.org/10.1016/j.scitotenv.2020.138884
16. Sy KTL, Martinez ME, Rader B, White LF (2020) Socioeconomic Disparities in Subway Use and COVID-19 Outcomes in New York City. American Journal of Epidemiology. https://doi.org/10.1093/aje/kwaa277

17. Takagi H, Kuno T, Yokoyama Y et al. (2021) Meta-regression of COVID-19 prevalence/fatality on socioeconomic characteristics of data from top 50 U.S. large cities. J Med Virol 93:595–598. https://doi.org/10.1002/jmv.26335
18. Whittle RS, Diaz-Artiles A (2020) An ecological study of socioeconomic predictors in detection of COVID-19 cases across neighborhoods in New York City. BMC Med 18:271. https://doi.org/10.1186/s12916-020-01731-6
19. Chen JT, Krieger N (2020) Revealing the Unequal Burden of COVID-19 by Income, Race/Ethnicity, and Household Crowding: US County Versus Zip Code Analyses. J Public Health Manag Pract 27. https://doi.org/10.1097/PHH.0000000000001263
20. Maroko AR, Nash D, Pavilonis BT (2020) COVID-19 and Inequity: a Comparative Spatial Analysis of New York City and Chicago Hot Spots. J Urban Health 97:461–470. https://doi.org/10.1007/s11524-020-00468-0
21. Liu SH, Liu B, Li Y, Norbury A (2020) Time courses of COVID-19 infection and local variation in socioeconomic and health disparities in England. medRxiv. https://doi.org/10.1101/2020.05.29.20116921
22. Baena-Díez JM, Barroso M, Cordeiro-Coelho SI, Díaz JL, Grau M (2020) Impact of COVID-19 outbreak by income: hitting hardest the most deprived. J Public Health (Oxf) 42:698–703. https://doi.org/10.1093/pubmed/fdaa136
23. Das A, Ghosh S, Das K, Basu T, Das M, Dutta I (2020) Modeling the effect of area deprivation on COVID-19 incidences: a study of Chennai megacity, India. Public Health 185:266–269. https://doi.org/10.1016/j.puhe.2020.06.011
24. Lamb MR, Kandula S, Shaman J (2020) Differential COVID-19 case positivity in New York City neighborhoods: Socioeconomic factors and mobility. Influenza Other Respir Viruses. https://doi.org/10.1111/irv.12816
25. Lieberman-Cribbin W, Tuminello S, Flores RM, Taioli E (2020) Disparities in COVID-19 Testing and Positivity in New York City. Am J Prev Med 59:326–332. https://doi.org/10.1016/j.amepre.2020.06.005
26. Rozenfeld Y, Beam J, Maier H et al. (2020) A model of disparities: risk factors associated with COVID-19 infection. Int J Equity Health 19:126. https://doi.org/10.1186/s12939-020-01242-z
27. Chang S, Pierson E, Koh PW et al. (2020) Mobility network models of COVID-19 explain inequities and inform reopening. Nature. https://doi.org/10.1038/s41586-020-2923-3
28. Moise IK (2020) Variation in Risk of COVID-19 Infection and Predictors of Social Determinants of Health in Miami-Dade County, Florida. Prev Chronic Dis 17:E124. https://doi.org/10.5888/pcd17.200358
29. Ginsburgh V, Magerman G, Natali I (2021) COVID-19 and the role of inequality in French regional departments. Eur J Health Econ. https://doi.org/10.1007/s10198-020-01254-0
30. Wachtler B, Michalski N, Nowossadeck E et al. (2020) Sozioökonomische Ungleichheit im Infektionsrisiko mit SARS-CoV-2 – Erste Ergebnisse einer Analyse der Meldedaten für Deutschland. Journal of Health Monitoring 5:19–31. https://doi.org/10.25646/7056
31. Plümper T, Neumayer E (2020) The Pandemic Predominantly Hits Poor Neighbourhoods? SARS-CoV-2 Infections and Covid-19 Fatalities in German Districts. Eur J Public Health. https://doi.org/10.1093/eurpub/ckaa168
32. Abedi V, Olulana O, Avula V et al. (2020) Racial, Economic, and Health Inequality and COVID-19 Infection in the United States. J Racial Ethn Health Disparities. https://doi.org/10.1007/s40615-020-00833-4
33. Mukherji N (2020) The Social and Economic Factors Underlying the Incidence of COVID-19 Cases and Deaths in US Counties. medRxiv. https://doi.org/10.1101/2020.05.04.20091041

34. Khanijahani A (2021) Racial, ethnic, and socioeconomic disparities in confirmed COVID-19 cases and deaths in the United States: a county-level analysis as of November 2020. Ethn Health 26:22–35. https://doi.org/10.1080/13557858.2020.1853067
35. Mourad A, Turner NA, Baker AW et al. (2020) Social Disadvantage, Politics, and SARS-CoV-2 Trends: A County-Level Analysis of United States Data. Clin Infect Dis. https://doi.org/10.1093/cid/ciaa1374
36. Richmond HL, Tome J, Rochani H, Fung IC-H, Shah GH, Schwind JS (2020) The Use of Penalized Regression Analysis to Identify County-Level Demographic and Socioeconomic Variables Predictive of Increased COVID-19 Cumulative Case Rates in the State of Georgia. Int J Environ Res Public Health 17. https://doi.org/10.3390/ijerph17218036
37. Palacio A, Tamariz L (2020) Social Determinants of Health Mediate COVID-19 Disparities in South Florida. J Gen Intern Med. https://doi.org/10.1007/s11606-020-06341-9
38. Chadeau-Hyam M, Bodinier B, Elliott J et al. (2020) Risk factors for positive and negative COVID-19 tests: a cautious and in-depth analysis of UK biobank data. Int J Epidemiol 49:1454–1467. https://doi.org/10.1093/ije/dyaa134
39. McQueenie R, Foster HME, Jani BD et al. (2020) Multimorbidity, polypharmacy, and COVID-19 infection within the UK Biobank cohort. PLOS ONE 15:e0238091. https://doi.org/10.1371/journal.pone.0238091
40. Ho FK, Celis-Morales CA, Gray SR et al. (2020) Modifiable and non-modifiable risk factors for COVID-19: results from UK Biobank. medRxiv. https://doi.org/10.1101/2020.04.28.20083295
41. Vahidy FS, Nicolas JC, Meeks JR et al. (2020) Racial and ethnic disparities in SARS-CoV-2 pandemic: analysis of a COVID-19 observational registry for a diverse US metropolitan population. BMJ Open 10:e039849. https://doi.org/10.1136/bmjopen-2020-039849
42. Blumenshine P, Reingold A, Egerter S, Mockenhaupt R, Braveman P, Marks J (2008) Pandemic influenza planning in the United States from a health disparities perspective. Emerg Infect Dis 14:709–715. https://doi.org/10.3201/eid1405.071301
43. Millett GA, Jones AT, Benkeser D et al. (2020) Assessing differential impacts of COVID-19 on black communities. Ann Epidemiol 47:37–44. https://doi.org/10.1016/j.annepidem.2020.05.003
44. Khansa Ahmad, Sebhat Erqou, Nishant Shah et al. (2020) Association of poor housing conditions with COVID-19 incidence and mortality across US counties. PLOS ONE 15:e0241327. https://doi.org/10.1371/journal.pone.0241327
45. Möhner M, Wolik A (2020) Berufs- und branchenbezogene Unterschiede im COVID-19-Risiko in Deutschland. Deutsches Ärzteblatt international 117:641–642
46. Gaudecker H-M von, Holler R, Janys, L., et (2020) Labour Supply in the Early Stages of the COVID-19 Pandemic: Empirical Evidence on Hours, Home Office, and Expectations. Zugegriffen: 3. März 2021
47. McCormack LA, Squiers L, Frasier AM, Lynch M, Bann CM, MacDonald PDM (2021) Gaps in Knowledge About COVID-19 Among US Residents Early in the Outbreak. Public Health Rep 136:107–116. https://doi.org/10.1177/0033354920970182
48. Williamson EJ, Walker AJ, Bhaskaran K et al. (2020) Factors associated with COVID-19-related death using OpenSAFELY. Nature 584:430–436. https://doi.org/10.1038/s41586-020-2521-4
49. Manabe T, Higuera Iglesias AL, Vazquez Manriquez ME et al. (2012) Socioeconomic factors influencing hospitalized patients with pneumonia due to influenza A(H1N1)pdm09 in Mexico. PLOS ONE 7:e40529. https://doi.org/10.1371/journal.pone.0040529
